# Respiratory cycle entrainment of septal neurons mediates the fast coupling of sniffing rate and hippocampal theta rhythm

**DOI:** 10.1111/ejn.12449

**Published:** 2014-08-20

**Authors:** Marian Tsanov, Ehsan Chah, Richard Reilly, Shane M O∼Mara

**Affiliations:** 1 Trinity College Institute of Neuroscience, Trinity College DublinDublin, 2, Ireland; 2 School of Psychology, Trinity College DublinDublin, Ireland; 3 Trinity Centre for Bioengineering, Trinity College DublinDublin, Ireland

**Keywords:** hippocampus, medial septum, respiration, theta rhythm

## Abstract

Memory for odour information may result from temporal coupling between the olfactory and hippocampal systems. Respiration defines the frequency of olfactory perception, but how the respiratory rate affects hippocampal oscillations remains poorly understood. The afferent connectivity of the medial septum/diagonal band of Broca complex (MS/DB) proposes this region as a crossroads between respiratory and limbic pathways. Here we investigate if the firing rates of septal neurons integrate respiratory rate signals. We demonstrate that approximately 50% of MS/DB neurons are temporally correlated with sniffing frequency. Moreover, a group of slow-spiking septal neurons are phase-locked to the sniffing cycle. We show that inter-burst intervals of MS/DB theta cells relate to the sniff rate. Intranasal odour infusion evokes sniff phase preference for the activity of fast-spiking MS/DB neurons. Concurrently, the infusion augments the correlation between sniffing and limbic theta oscillations. During periods of sniffing–theta correlation, CA1 place cells fired preferentially during the inhalation phase, suggesting the theta cycle as a coherent time frame for central olfactory processing. Furthermore, injection of the GABAergic agonist muscimol into medial septum induces a parallel decrease of sniffing and theta frequencies. Our findings provide experimental evidence that MS/DB does not merely generate theta rhythm, but actively integrates sensorimotor stimuli that reflect sniffing rate. Such integration may provide temporal oscillatory synchronisation of MS/DB-innervated limbic structures with the sniffing cycle.

## Introduction

One of the main sources of information for rodents about the surrounding environment is provided by the olfactory system. Olfactory perception has two components: (i) motor – the generation of fast respiratory rhythm (sniffing), and (ii) sensory – the decoding of odour stimuli. Activity in the olfactory bulb (OB) is periodically modulated by the animal∼s respiration; the phasic activity of bulbar neurons is dependent on nasal airflow (Ravel *et al*., 1987; Charpak *et al*., 2001; Spors & Grinvald, 2002; Buonviso *et al*., 2003; Scott, 2006). Thus, the rate of olfactory perception depends on the generation of respiratory rhythm.

Tagging odours to locations may be an important mechanism by which rodents navigate (Eichenbaum *et al*., 2007; Robitsek *et al*., 2008). The hippocampus encodes spatial representations of the environment, and changes in hippocampal physiology have also been correlated with respiration (Kay, 2005). The relationship between hippocampal theta rhythm (6–12 Hz) and automatic behaviours such as whisking and sniffing has been continuously debated (Kepecs *et al*., 2006). The current understanding is that theta and whisking/sniffing are independent oscillators, which may synchronise in the presence of a strong sensorimotor stimulus (Berg *et al*., 2006; Buonviso *et al*., 2006). Theta rhythm correlates with sniffing during odour contingency reversal learning (Macrides *et al*., 1982). Hippocampal theta also intermittently couples with OB oscillations during exploratory behaviour (Vanderwolf, 1992). The firing of single interneurons in the hippocampus is linked to performance in an olfactory identification task (Wiebe & Staubli, 2001). Thus, theta oscillation might provide the timing frequency of respiratory and limbic integration (Kepecs *et al*., 2006). On a systems level, however, there are different theories suggesting how these activities are synchronised and the network of centrifugal olfactory processing remains largely unexplored (Wachowiak, 2011).

Anatomical and physiological evidence suggest the medial septum/diagonal band of Broca complex (MS/DB, the target of our investigation here) as the key structure involved in the integration of respiration rate and limbic theta oscillations. First, the MS/DB receives ascending projections from cholinergic neurons in the laterodorsal and pedunculopontine tegmental nuclei (pontine respiratory group) (Fibiger, 1982; Satoh & Fibiger, 1986; Jones & Beaudet, 1987; Woolf & Butcher, 1989). The same pontine nuclei send descending inputs to the dorsal and ventral respiratory groups (Jones, 1990; Semba & Fibiger, 1992; Kubin & Fenik, 2004) that generate the respiratory rhythm (Smith *et al*., 1991; Onimaru & Homma, 2003). Second, GABAergic and cholinergic neurons in MS/DB play a crucial role in the regulation of hippocampal activity (Vertes & Kocsis, 1997; Buzsáki, 2002).

In turn, MS/DB efferents diverge across several limbic structures, where they regulate the amplitude and frequency of local field theta oscillations. Theta rhythm entrains hippocampal pyramidal neurons; the spiking rate of place cells relates to the phase of theta rhythm (O∼Keefe & Recce, 1993; Skaggs *et al*., 1996). In the present study, we performed single-unit extracellular recordings in MS/DB and hippocampal formation and evaluated neuronal activity in relation to the respiratory rate and phase in behaving rats. Here, we investigate if the septal region integrates olfactory sensorimotor processing and if inactivation of MS/DB reflects concurrent alterations in respiration and theta frequencies. We suggest that the coordination of hippocampal neurons with sensorimotor olfactory processing is mediated by MS/DB-generated theta rhythm.

## Materials and methods

### Surgical implantation of electrodes

The surgical implantation and the single-unit recordings were performed as previously described (Tsanov *et al*., 2011). Tetrodes were implanted in MS/DB (+0.5 AP, −1.1 ML, angle 10° medially and 5.5 mm dorsoventral to dura) and hippocampus (−3.8 AP, −2.3 ML and 1.8 mm dorsoventral to dura) of male Lister-Hooded rats (Harlan, UK). Experiments were conducted in accordance with European Community directive 86/609/EC and the Cruelty to Animals Act, 1876, and were approved by the Irish Medical Board (project authorisation number: AE19136/P003). The study followed Bioresources Ethics Committee, Trinity College Dublin, Ireland, and international guidelines of good practice.

### Measurement of sniffing

We implanted a 22-gauge hollow guide cannula (C313G; Plastics One, Roanoke, VA, USA) for intranasal pressure measurements (Verhagen *et al*., 2007). During the surgery, the intranasal cannula was lowered into the dorsal recess (frontal–nasal fissure: 0 mm AP, ML: 0.9 mm, angle toward midline: 4°) and fixed to the rest of the headstage with dental cement. After recovery, sniffing was continuously recorded throughout all recording sessions by connecting the guide cannula implanted in the dorsal recess to a pressure sensor (0-1PSIG 24PCAFA6G; Farnell Ltd, Leeds, UK) via PE tubing (0.45 m × 0.1 mm ID × 0.15 mm OD). Sensor output was amplified 100×, low-pass filtered at 100 Hz, connected to the preamplifier and synchronised with tetrode recordings.

### Recording techniques

After at least 1 week∼s recovery, subjects were connected, via a 32-channel headstage (Axona Ltd, St Albans, UK), to a recording system, which also allowed for animal position tracking. Signals were amplified (× 10000–30000) and band-pass filtered between 380 Hz and 6 kHz for single-unit detection. Candidate waveforms were discriminated off-line using graphical cluster-cutting software (Axona), which allows waveform separation based on multiple features including spike amplitude, spike duration and spike voltage. Autocorrelation histograms were calculated for each unit, and the unit was removed from further analysis if the histogram revealed the existence of correlations within the first 2 ms (refractory period), inconsistent with good unit isolation. Local field potential (LFP) recordings and analysis were performed as previously described (Tsanov *et al*., 2011). We measured LFP from the tetrode wires with the lowest impedance (50–250 KΩ) and for LFP analysis we used a band-pass filter (< 15 Hz).

### Recording sessions

The recordings took place in a square arena (64 × 64 × 25 cm high) and/or rectangle-shaped linear track (64 × 64 × 9.5 cm and wide) situated in the centre of a room with multiple background cues available. Rats were placed in the open field and 20-mg food pellets (Formula 5TUL; TestDiet, St Louis, MO, USA) were thrown in every 20 s to random locations within the open field; in this way, animals locomoted continuously, allowing for complete sampling of the environment. The duration of each experimental session was 16 min.

### Criteria for data inclusion

Cells were selected on the basis of their firing frequency, rhythmicity of their firing and spike isolation. We identified 334 well-isolated units within the MS/DB from seven rats and 157 well-isolated units within hippocampal area CA1 from five rats (250–350 g). Based on post-mortem histological criteria, we estimated that 150 of these units were from medial septum, and 184 were from diagonal band of Broca. Units from MS/DB with mean firing frequency < 10 Hz were classified as slow-spiking, and with mean firing frequency > 10 Hz as fast-spiking. Bursting units with an autocorrelogram theta index value of > 0.1 were classified as theta units (Tsanov *et al*., 2011). Bursts were defined as a sequence of three or more spikes with an inter-spike interval of 4 ms (Ramcharan *et al*., 2005). Spike trains were defined as a sequence of three or more spikes with an inter-spike interval of < 10 ms and a 15- to 250-ms pause, preceding the first spike of the train (Tsanov *et al*., 2011). Single hippocampal pyramidal cells and interneurons were identified using spike shape and firing frequency characteristics (Wilson & McNaughton, 1993; Csicsvari *et al*., 1999). Place fields were identified as described before (Wang *et al*., 2012). The firing map reflected the frequency of firing calculated from the top firing bins down to the bins with lowest firing rate by scaling them in decreasing intervals of 20% of the peak firing (Hollup *et al*., 2001; Brun *et al*., 2002).

### Phase analyses

Each spike was assigned a position on the sniffing phase and/or theta phase (band-pass filtered LFP for CA1 recordings) between 0 and 360° (Mehta *et al*., 2002; Siapas *et al*., 2005). The peaks of the sniffing or theta signals were detected using the gradient for each cycle (defined as the signal between two successive peaks). A sine wave was fitted to the signal using the least square method. This fitted sine wave was then used to estimate the phase of sniffing or theta signal at each sample point, similar to previous procedures (O∼Keefe & Recce, 1993). Rayleigh∼s test of uniformity was used to test phase distributions for deviations from the circular uniform distribution (Fisher, 1993). The circular concentration coefficient, kappa *k*, was also employed to quantify the phase locking (Siapas *et al*., 2005). Higher values of *k* correspond to narrower distributions around a mean preferred phase (Jones & Wilson, 2005). The *k* value was calculated using Matlab circular statistics tool box (http://philippberens.wordpress.com/code/circstats/). Phase precession of place cells in CA1 on rectangle-shaped linear tracks was analysed over the length of a cell∼s place field. As such, the field size imposes natural bounds on the times and positions over which precession can occur. Linear regression was used to quantify the position vs. phase correlations that arise as a consequence of phase precession, calculated using Pearson∼s correlation coefficient (*r*^2^).

### Correlation analysis

We used similarity measure analysis (Lyttle & Fellous, 2011) to examine the correlation between inter-spike intervals and sniff cycles. We performed a correlation analysis between inter-spike distance and the timing between two sniff cycles, using a modification of the similarity measuring method (Lyttle & Fellous, 2011). The sniffing signal was transformed in series of sine waves, and the entire signal was represented by their phase. This approach transforms the signal from a sine waveform to series of functions that linearly increase between sniffing cycles. Similarly, spike timing is transformed in a series of function that increase linearly with time (Lyttle & Fellous, 2011). Assuming a spike train with timings *d* = *d*_1_, *d*_2_, … *d*_*n*_, where *n* is the number of spikes detected for the cell during the recording, the transformation *g*(*t*) is, where *t* represents time:


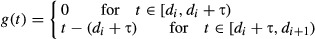


We selected the maximum inter-spike interval that considers two spikes as one event, τ at 10 ms, to ascertain that spike trains are considered as one event. Finally, Pearson correlations were calculated between both signals at time lags to determine the inter-spike intervals and sniffing cycle relationship. A value of one at time zero would indicate a perfect relationship between the two signals.

The peak values of the cross-correlograms are positive when the spikes correlate with the peaks of the respiration cycle (0/360°), negative when the spikes correlate to the troughs of the respiration cycle (180°) and when the spikes that correlate with degrees from the descending (90°) or ascending (270°) slope of the cycle the peak values contain negative and positive components.

### Coherence analyses

We calculated the coherence between sniff and the hippocampal LFP, using NeuroSpec (Halliday *et al*., 1995), version 2.0 for MATLAB. In the time domain, the cumulant density function was estimated from the cross-spectrum, via Fourier transform. The coherence uses the method of disjoint sections, where the recording (*R*) is divided into *L* non-overlapping epochs, each with time length *T*, where *R* = *LT*. The time length of *T* is approximately 2 s and *T* is equal to 512 samples, while *R* is the length of the analysed recoding, i.e. 16 min. For the segmented signal *x*, Discrete Fourier Transforms is used to estimate the auto-spectrums of the signals *f*_*xx*_(λ), where λ is a particular frequency. The linear relationship (coherence) between signals *x* and *y* is *R*_*xy*_, which is estimated using auto and cross spectrums:


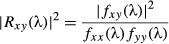


### Shuffling spike analysis

To test the statistical significance of phase preference of neuronal spiking activity, we performed a spiking shuffle analysis (Louie & Wilson, 2001; Brandon *et al*., 2012). The spike times were divided into bins of 0.004 s, corresponding to the sampling frequency of LFP and sniffing signals. These spiking times were then randomly permutated to remove temporal alignment while maintaining the overall activity of the cell. The cell recording was then compared with the maximal random shuffled value.

We calculated the normal distribution of the firing frequency of the recorded neurons as a function the sniff phase (0–360°). For this the phase was binned into segments with bin size of 20°, resulting in 18 phase bins. One thousand permutations were used to estimate the firing frequency for all bins with mean value and standard deviation. The Z-score of the recorded spikes was calculated by subtracting the mean shuffled frequency from the recorded firing frequency and dividing over the shuffled standard deviation. The threshold z score of α level 1.96 corresponds to a probability of less than 5% and expresses the statistical significance for phase preference. Therefore, if *Z* ≥ 1.96 or ≤ −1.96, the probability that the observed firing frequency is consistently phase-locked is ≤ 0.05.

### Odor delivery

Saturated odour vapour was produced by flowing clean (carbon-filtered) air through disposable syringe filters which were loaded with 30 μL liquid odorant (Verhagen *et al*., 2007). The flow rate of odorant streams (vanilla scent) was maintained at level of 50 mL/min, and a variable air flow dilution of 5% was produced.

### Pharmacological manipulation

Muscimol, a GABA(A) agonist, was diluted in phosphate-buffered saline (PBS) to 0.5 μg/mL (Shirvalkar *et al*., 2010; Brandon *et al*., 2011). Prior to an infusion, the dummy cannula was removed to allow access to the guide cannula. A unilateral injector cannula, cut to a length 1 mm longer than the guide cannula, was filled with the muscimol solution and lowered through the guide cannula and into the MS/DB. A microinfusion pump infused 0.5 μL of muscimol solution at 0.1 μL/min. The injector cannula remained connected for 1 min after the infusion to allow for the drug to perfuse through the neural tissue. The injector was removed and a dummy cannula was secured to the guide cannula. Recordings were continued 15 min after muscimol injection. The animals received a muscimol injection only once. At the end of the study brains were removed for histological verification.

### Statistical analyses

All data were analysed using Prism software (GraphPad Software, La Jolla, CA, USA). Statistical significance was estimated by using a two-tailed *t*-test, non-parametric Watson–Williams test and two-way analysis of variance (anova) paired with *post hoc* Newman–Keuls test. The probability level interpreted as statistically significant was *P <* 0.05. All data points are given as mean ± SEM.

## Results

**Fig. 1:**
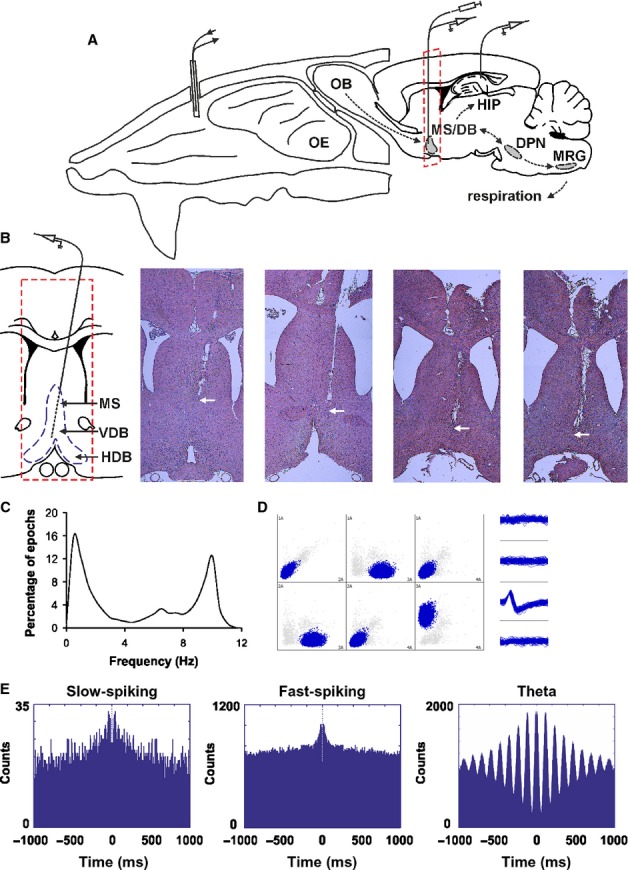
Anatomical and electrophysiological identification of septal activity. (A) Atlas schematic of our experimental set-up investigating the functional relation of septal region to respiratory circuitry in freely behaving rats (OE, olfactory epithelium; OB, olfactory bulb; HIP, hippocampus; MS/DB, medial septum/diagonal band of Broca; DPN, dorsal pontine nuclei, including laterodorsal and pedunculopontine tegmental nuclei; MRG, medullar dorsal and ventral respiratory groups). Nasal implant allowed measurement of intranasal pressure and/or odorant infusion. Microdrive implant allowed measurement of single unit activity in MS/DB and in hippocampal area CA1. A cannula was also implanted in MS/DB for muscimol injections. The red rectangle denotes the plane of histological sections below. (B) Coronal atlas schematic (left) and histological sections from four rats where eight tetrodes were implanted in medial septum and subsequently lowered into the diagonal band of Broca. The white arrow indicates the location of the tetrodes∼ tip (VDB, HDB, vertical and horizontal limbs of diagonal band). (C) Frequency histogram of sniffing in behaving animals during pellet-chasing task. (D) Sample scatterplot, showing all signals recorded between each pair of electrodes on a given tetrode. Sample waveform (right) of an MS/DB unit, corresponding to the blue cluster in the scatterplot. (E) Sample of 1000-ms autocorrelograms of the main neuronal groups: slow-spiking (left), fast-spiking (middle) and theta units (right).

To monitor sniffing activity, we implanted a pressure sensor in nasal dorsal medial meatus of 12 rats, allowing us to measure the pressure changes between inspired and expired air (Fig. 1A). In parallel, we implanted a microdrive with eight tetrodes that were lowered through the MS/DB (Fig. 1B). After 1 week of recovery, we recorded simultaneously respiration and the spiking activity of MS/DB in behaving animals (Fig. 1C and D). The recordings were performed in a square arena, which the animals explored during a pellet-chasing task (for 16 min). We identified 334 well-isolated units, histologically assigned to MS/DB (for regional distribution see Materials and methods), of which 175 (52%) fired in relation to the animal∼s sniffing frequency. To evaluate how spiking activity of the recorded units relates to sniffing, we performed two types of analyses: (i) distribution of the spikes throughout the sniffing phase (between 0 and 360°) and (ii) correlations between the inter-spike interval and the rate of sniffing cycles. We also analysed the polarity of spiking with respect to the main sniffing frequency bands (Fig. 1C). Based on their firing frequency and rhythmicity, we grouped the sniff-modulated units into slow-spiking, fast-spiking and theta groups (Fig. 1E).

### Phase-lock of slow-spiking MS/DB units to the sniff phase

**Fig. 2:**
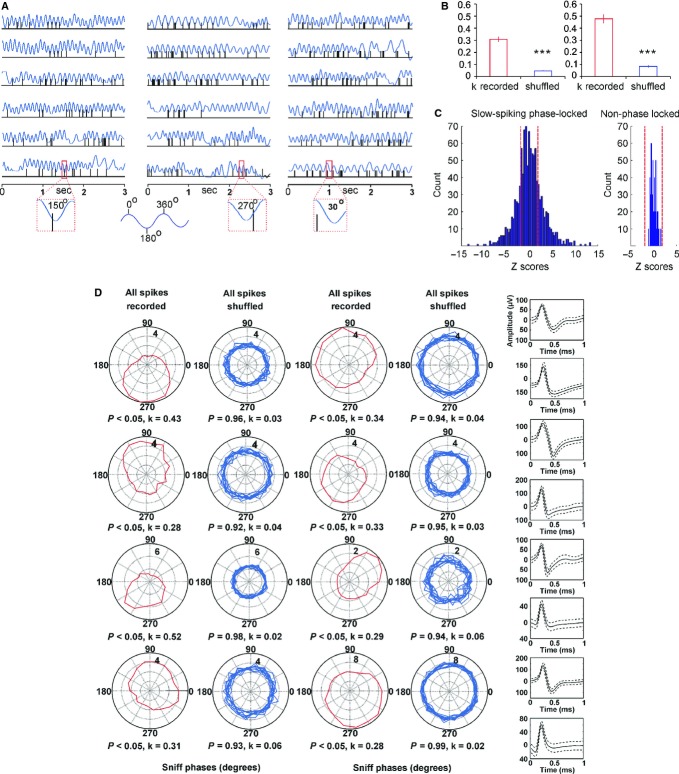
Phase-locking of slow-spiking MS/DB units to the sniff cycle. (A) Sample recordings of three slow-spiking units and concurrent sniffing (blue). The insets below amplify one sniffing cycle (marked with red rectangle) from each unit and show the polarity of the respective spike. Sniff peak represents 0/360°, while sniff trough −180°. (B) Left: average values of circular correlation coefficient *k* for recorded (red bar) and shuffled spikes (blue bar). Right: average *k* values compared between recorded (red bar) and shuffled spikes (blue bar) for sniffing frequency of 9–12 Hz. ***P < 0.001. (C) Distribution of Z-scores evaluates the significance of phase locking to the sniff cycle for slow-spiking units. Significance probability associated with Z shows the relationship of the neuronal firing frequency across all phase bins (0–360°). Dashed red lines indicate the *P* = 0.05 significance threshold, and thus all values of Z to the right/left of these lines are significant at that confidence level. Left: averaged distribution for all slow-spiking units; right: averaged distribution for septal units not associated with the sniff cycle. (D) Sample polar plots, showing the firing rate (Hz) (dotted circles), relative to the sniff cycle for eight slow-spiking units. The peak-to-peak distance of one sniff cycle is considered as 0–360° where the trough is 180° (see inset). Each panel shows the phase parameters for recorded spikes (red, left) and shuffled (blue, right) data. Right: spike waveforms of the slow-spiking group, characterized with a mean spike amplitude of 149.2 ± 25.9 μV and spike width of 145.9 ± 24.5 μs. Solid curve represents the mean and the dashed curve represents the standard deviation. Solid curve represents the mean and the dashed curve represents the standard deviation.

The slow-spiking units (64/19%) are cells with a low average firing frequency (3.1 ± 0.3 Hz, Fig. 2A). They were significantly phase-locked to the sniffing cycle, where each cell expressed an individual phase preference (Fig. 2D). The statistical significance of phase-locking was evaluated using Rayleigh∼s statistic (Fisher, 1993). We compared circular concentration coefficient *k* values from recorded spikes (Jones & Wilson, 2005; Siapas *et al*., 2005) to *k* values from the spikes when shuffled (Brandon *et al*., 2012) within time bins of 0.004 s (Fig. 2B, left). As expected, the shuffled spikes expressed no phase-preference, with *k* values significantly lower compared with recorded spikes (*P* < 0.001, Watson–Williams test). To estimate the null distribution of septal firing frequency across the sniff phase we used 1000 shuffles. After we found the upper 95% value we compared the recorded data using Z-scores (see Methods). For Z scores calculation the mean shuffled frequency of 1000 permutations was subtracted from the neuronal firing frequency for all degrees (with phase bin of 20°) and divided over the shuffled standard deviation. The threshold α level of 1.96 (marked with red dashed lines in Fig. 2) corresponds to a probability of less 5% and expresses the statistical significance for phase preference. Slow-spiking units show a high number of significant Z-scores (Fig. 2C, left). Concurrently, septal units with no phase preference show Z-scores only within the non-significant range (Fig. 2C, right).

**Fig. 3:**
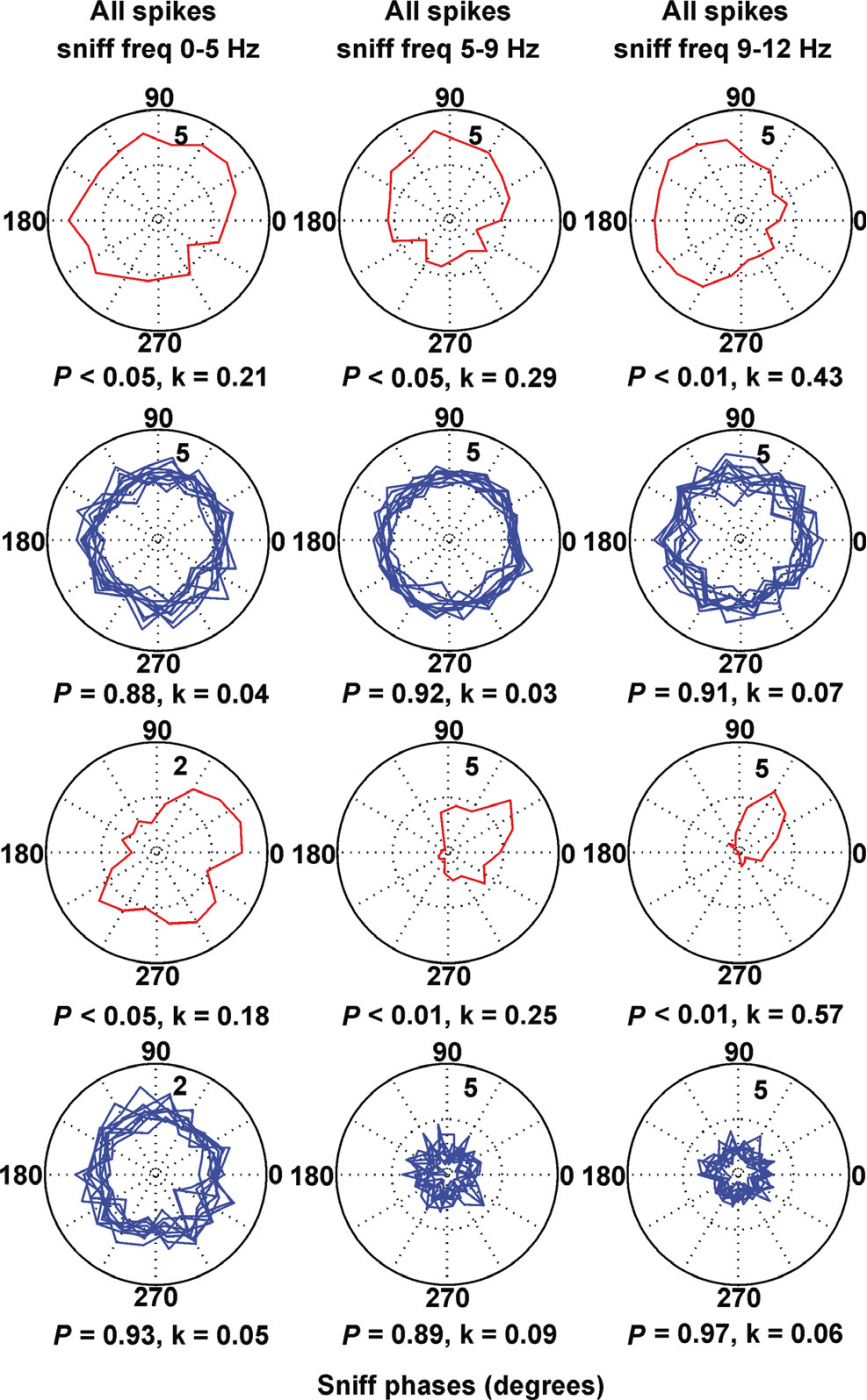
Frequency-dependent phase-preference of the slow-spiking units. Sample polar plots, relative to the sniff cycle for two slow-spiking units in relation to the sniffing frequency: left panels, 0–5 Hz; middle panels, 5–9 Hz; right panels, 9–12 Hz. Red traces represent recorded, while blue traces represent shuffled spikes.

The transition from resting respiration (0–5 Hz) to active odour sampling (5–12 Hz, typically termed ‘sniffing’) can occur in a single respiration cycle. As respiration rates show a continuous distribution throughout the recording session (Verhagen *et al*., 2007), we use ‘sniffing’ to refer to all respiratory activity regardless of frequency (Kepecs *et al*., 2006). Similar to previous findings (Kepecs *et al*., 2007), the frequency histogram from our recordings revealed three major sniffing frequency bands. We then analysed the polar distribution of the spikes across the frequencies in the low (0–5 Hz), intermediate (5–9 Hz) and high frequency range (9–12 Hz) (Fig. 3; Fig. S1). We found that the polarity in the range 9–12 Hz was characterised by the highest *k* values (0.47 ± 0.03, Fig. 2B, right), which are significantly higher (*P* < 0.05, Watson–Williams test) compared with the non-filtered (0–12 Hz) *k* values of the recorded spikes (0.31 ± 0.02).

### Temporal entrainment of fast-spiking and theta MS/DB activity to the sniffing cycle

**Fig. 4:**
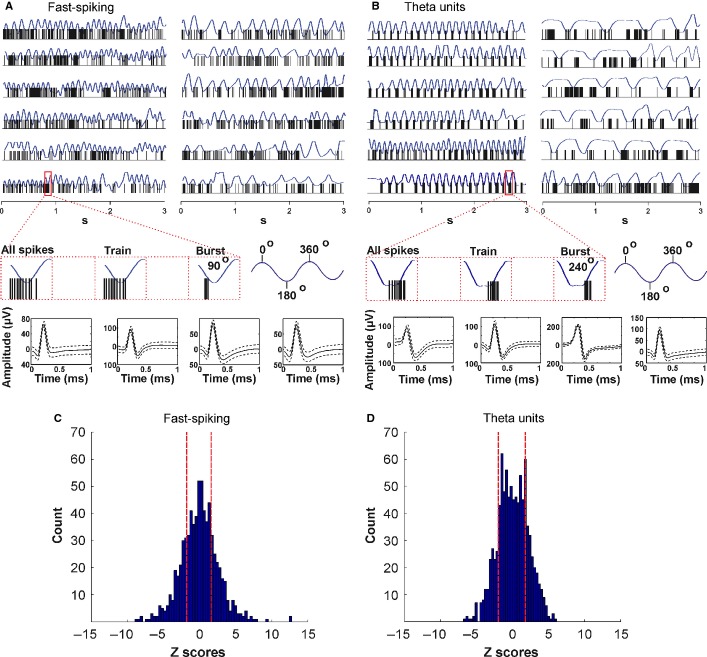
Firing patterns of fast-spiking and theta MS/DB units. (A) Sample recordings of a fast-spiking unit and concurrent sniffing (blue) during fast (left traces) and slow (right traces) sniff. The inset below amplifies one sniffing cycle (marked with red rectangle) and shows the polarity of the respective spikes (left), train (middle) and burst (right). Sniff peak represents 0/360°, while sniff trough −180°. Bottom: spike waveforms of four fast-spiking units. The fast-spiking group is characterised with mean spike amplitude of 145.7 ± 19.8 μV and spike width of 126.9 ± 15.8 μs. (B) Sample recordings of theta unit and concurrent sniffing (blue) during 9–12 Hz (left traces) and 0–5 Hz (right traces) sniffing frequency. The inset below amplifies one sniffing cycle (marked with red rectangle) and shows the polarity of the respective spikes (left), train (middle) and burst (right). Bottom: spike waveforms of four theta units. The theta group is characterised with a mean spike amplitude of 126.9 ± 25.3 μV and spike width of 115.8 ± 16.7 μs. The distribution of Z-scores evaluates the significance of phase locking to the sniff cycle for fast-spiking units (C) and theta units (D). Significance probability associated with Z shows the relation of the neuronal firing frequency across all phase bins (0–360°). Dashed red lines indicate the *P* = 0.05 significance threshold, and thus all values of Z to the right/left of these lines are significant at that confidence level.

We next analysed the group of fast-spiking units (45/13%), characterised by a high average firing frequency (26.3 ± 2.2 Hz), and the group of septal ‘theta’ cells (66/20%; average firing frequency 35.5 ± 2.8 Hz). Unlike for the slow-spiking units, the relation to the sniffing cycle of fast-spiking units is less obvious when visualised on raw data traces (Fig. 4A). These neurons tend to group their spikes in clusters and, in addition, they fire in bursts. We examined separately the bursts with an inter-spike interval ≤ 4 ms and the spike trains with an inter-spike interval of ≥ 4 ms and ≤ 10 ms (Fig. 4A, red-marked inset).

Theta cells are the most representative cell type of the medial septum (Ranck, 1973; Stewart & Fox, 1989b; King *et al*., 1998), related to its functional role as a theta rhythm generator (Bland & Bland, 1986; Stewart & Fox, 1989a). The main feature of theta cells in regard to sniffing is their ability to burst with a frequency similar to the sniffing rate for several seconds (Fig. 4B, left). During epochs with slow sniffing (0–5 Hz), characterised behaviourally by lower levels of arousal, theta units disengage from their rhythmic bursting, thereby reducing the degree of coupling with the sniff rate (Fig. 4B, right). We also analysed their activity for all spikes, spike trains and bursts (Fig. 4B, red-marked inset). Z-score analysis of fast-spiking (Fig. 4C) and theta units (Fig. 4D) shows that the firing frequency of both cell types expresses a sufficient number of Z-scores above the α level threshold of 1.96, corresponding to a probability of more than 95% for phase preference. Although the number of significant counts was less compared with the slow-spiking units the observed values reveal that a fraction of the fast spiking and theta firing frequency is consistently phase-locked to the sniff cycle.

**Fig. 5:**
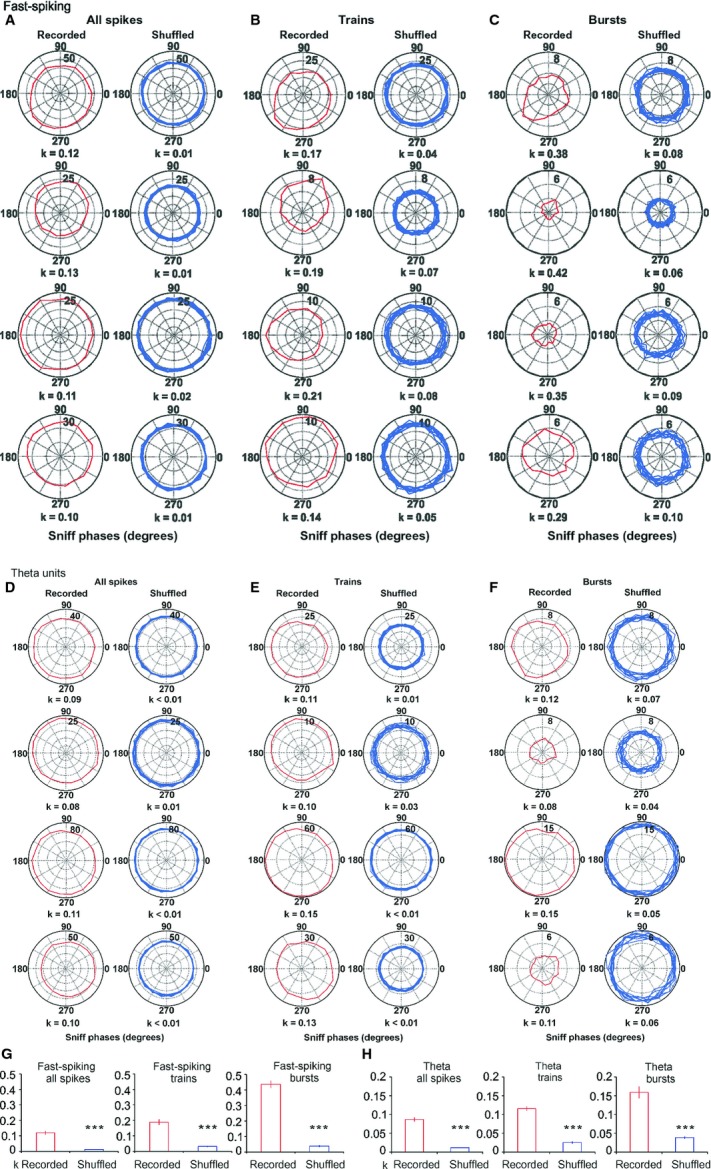
Sniff modulation of fast-spiking and theta MS/DB units. (A) Sample polar plots, showing firing rate (Hz) (dotted circles), relative to the sniff cycle for four fast-spiking units. Each panel shows the phase parameters for recorded spikes (red, left) and shuffled (blue, right) data. (B) Sample polar plots, relative to the sniff cycle for the same units for spike trains. (C) Sample polar plots, relative to the sniff cycle for the same units for bursts. Red traces represent recorded, while blue traces represent shuffled spikes. (D) Sample polar plots, showing firing rate (Hz) (dotted circles), relative to the sniff cycle for four theta units. Each panel shows the phase parameters for recorded spikes (red, left) and shuffled (blue, right) data. (E) Sample polar plots, relative to the sniff cycle for the same units for spike trains. (F) Sample polar plots, relative to the sniff cycle for the same units for bursts. Red traces represent recorded, while blue traces represent shuffled spikes. (G) Fast-spiking average values of circular correlation coefficient *k* for recorded (red bars) and shuffled spikes (blue bars) for all spikes (left plot), spike trains (middle) and bursts (right). ***P < 0.001. (H) Theta average values of circular correlation coefficient *k* for recorded (red bars) and shuffled spikes (blue bars) for all spikes (left plot), spike trains (middle) and bursts (right). ***P < 0.001.

The phase polarity of fast-spiking cells was less well expressed and *k* showed smaller values for all recorded spikes, compared with slow-spiking units (Fig. 5A and G, left). The distribution of the spike trains and bursts across the sniff cycle was phase-dependent (Fig. 5B and C), with higher *k* values (0.19 ± 0.02 for spike trains and 0.43 ± 0.01 for bursts, Fig. 5G middle and right, respectively) compared with shuffled spikes (*P* < 0.001, Watson–Williams test). Thus, spike trains/bursts define the relation of this group of cells to the sniffing cycle. Another difference between fast-spiking units and slow-spiking units is the lack of a phase-lock preference for the high theta sniffing range (9–12 Hz). A similar degree of phase polarity is expressed for the three sniff frequency ranges (0.11 ± 0.07 for 0–5 Hz; 0.10 ± 0.12 for 5–9 Hz; 0.14 ± 0.09 for 9–12 Hz; Fig. S2). Theta cells show a spike preference with the sniff phase, with *k* values for all recorded spikes significantly higher than the shuffled spikes (*P* < 0.001, Watson–Williams test, Fig. 5D and H, left). Although theta spike trains and bursts (Fig. 5E and F) revealed even higher *k* values (0.11 ± 0.01 and 0.16 ± 0.02, respectively; Fig. 5H middle and right), their phase polarity was less prominent compared with the slow- and fast-spiking units (Fig. S3).

### Correlation analysis of septal spiking and sniffing rate

**Fig. 6:**
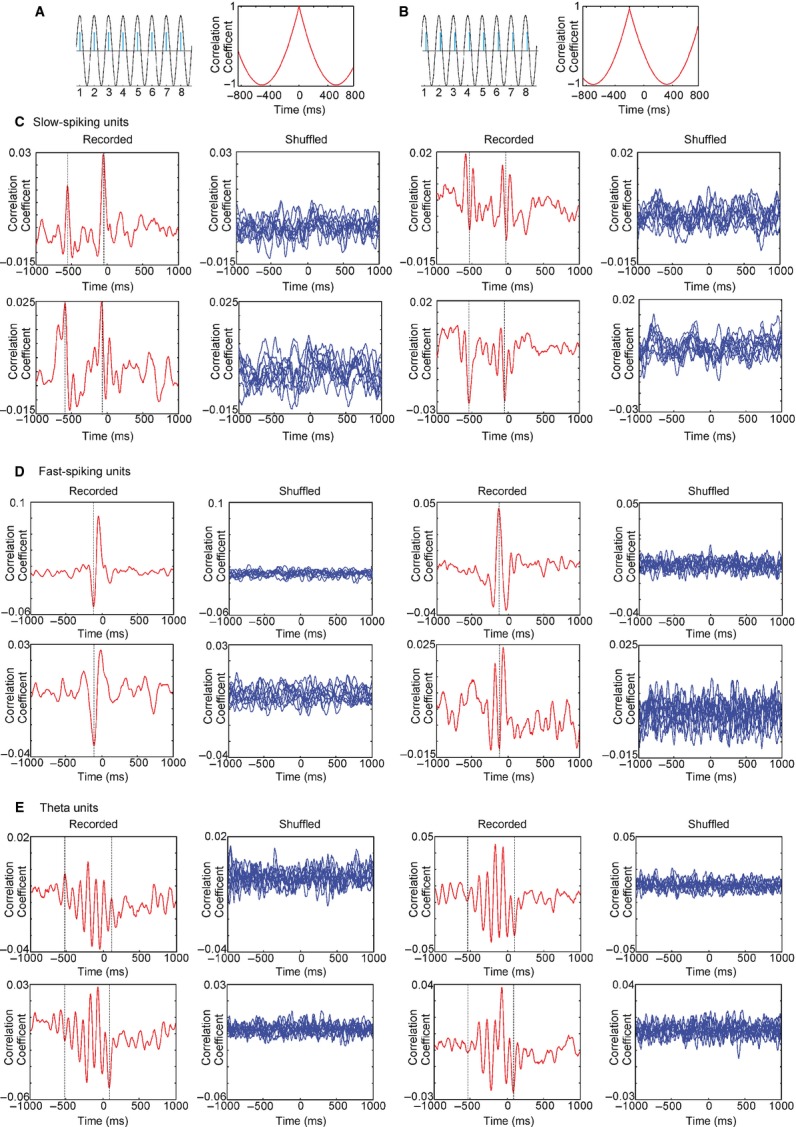
Phase relationship between septal cell types and sniff cycle. (A) Inter-spike correlation between sinusoid oscillation (black) and modelled spikes (blue) when the oscillation and spikes are in-phase. Cross-correlogram example (right panel) shows the peak of correlation at time 0. (B) Cross-correlogram example for phase-shifted spikes shows the peak of correlation in the negative part of the *x*-axis, left from time 0 (right panel). (C) Sample cross-correlograms of recorded (red, left) and shuffled (blue, right) spikes from four slow-spiking units. Dashed lines on each panel represent positive/negative correlation peaks. (D) Sample cross-correlograms of recorded (red, left) and shuffled (blue, right) spikes from four fast-spiking units. The dashed line on each panel represents a time lag of −100. (E) Sample cross-correlograms of recorded (red, left) and shuffled (blue, right) spikes from four theta units. Dashed lines represent a time lag of +100 (right line) and −550 (left line).

Another approach to detecting parallel patterns between neuronal spiking and an oscillation is to correlate the inter-spike intervals with the duration of oscillatory cycles. This correlation reveals if there is a consistent temporal match between these parameters and the time-shift between them (Fig. 6A and B). We correlated inter-spike intervals of septal neurons with sniffing, and compared the result with the correlation of the shuffled spikes and sniffing cycles. We found a peak in the cross-correlogram for the recorded spikes (Fig. 6C, red), but not for the shuffled spikes (Fig. 6C, blue). Interestingly, the slow-spiking units were characterised by a second peak at about −500 ms (with regard to the *x*-axis). Both correlation peaks were shifted by −30 ± 10 ms from time 0 and time lag −500, respectively. Thus, the inter-spike intervals are shifted from 20 to 40 ms, by consistently matching sniff cycles. Importantly, slow-spiking neurons repeat this correlated firing pattern after 500 ms. This characteristic feature suggests that the slow-spiking units correlate their rate with the sniffing every 4–5 cycles on average. The correlation peaks of different neurons revealed no preference for the sign of correlation, which can be negative or positive. The mean value of the correlation peaks for all cases (0.021 ± 0.003) was significantly higher (*P* < 0.01, *t*-test) than the mean correlation peak for shuffled spikes (0.010 ± 0.002). The interval of about 30 ms between lag 0 and the first correlation peak shows that the slow-spiking units are modulated by respiration with an oligosynaptic time delay.

The correlation between the inter-spike interval and the sniffing cycle revealed a characteristic profile of the fast-spiking units (Fig. 6D). It shows a single negative/positive peak, which was shifted to −100 ± 10 ms from time lag 0. Therefore, the inter-spike intervals (which are also set by the spike trains and bursts) of these neurons are preceded by 90–110 ms by the corresponding sniff rate. The peaks revealed high correlation values (0.034 ± 0.005), compared with the shuffled spikes correlation values (0.011 ± 0.003) (*P* < 0.001, *t*-test). The ability of theta cells to burst with a frequency similar to the sniffing rate is expressed through the higher values of the inter-spike-sniff correlation (0.038 ± 0.007). Furthermore, the correlation between inter-spike intervals and sniff cycles resulted in multiple positive and negative peaks (Fig. 6E). The distance between two positive (or two negative) peaks was in the range 80–130 ms, reflecting the bursting frequency of theta cells (8–12 Hz). The oscillation of correlation peaks ranged between +100 and −550 ms. This result suggests that theta bursting activity couples with the sniffing rate for about 0.5 s on average. The presence of several correlation peaks on both sides of lag 0 suggests that theta units integrate intrinsic burst oscillations with extrinsic oscillations of sniffing rate. The positioning of the majority of peaks within the negative part of the *x*-axis (left from time lag 0) reveals that the sniff itself initiates the coupling of both oscillations.

### Olfaction-induced sniff-phase spiking preference of septal neurons parallels correlations between the sniff rate and the hippocampal theta rhythm

**Fig. 7:**
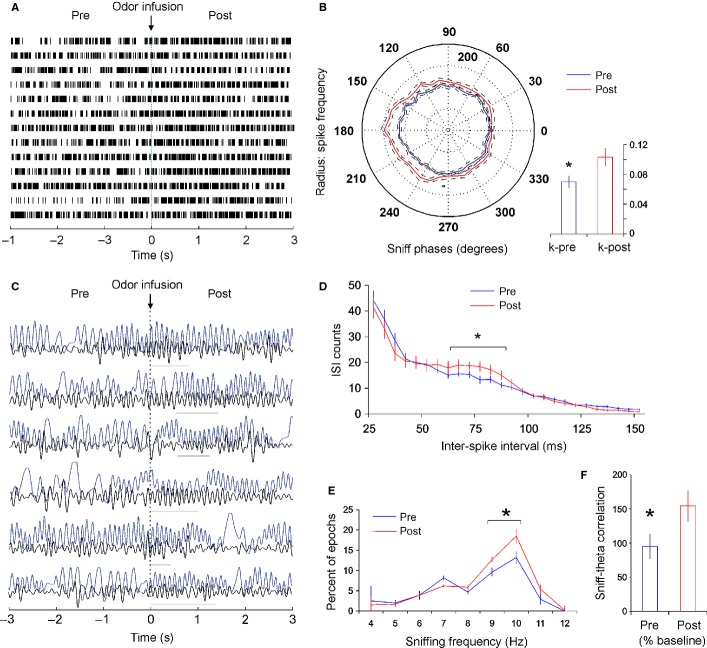
Odour-stimulation augments sniff-modulated spiking and increases the correlation between sniff and hippocampal theta. (A) Sample recordings of a fast-spiking unit before and after odour infusion. The dotted vertical line at time 0 indicates the time of infusion. (B) Average polar plot, showing normalised firing rate (%), relative to the sniff cycle for all fast-spiking units (mean ± SEM). The peak of sniff polarity for all units is normalised to 180°. Inset below, right: average *k* values compared between pre- (blue bar) and post-infusion spikes (red bar). (C) Sample recordings of local field potential (LFP, low band-pass filtered) and concurrent sniffing (blue) before and after odour infusion. The dotted vertical line at time 0 indicates the time of infusion. Horizontal grey lines indicate epochs of high correlation between both oscillations. (D) Inter-spike histogram of the spike intervals before (blue) and after (red) odour infusion. *P < 0.05. (E) Frequency histogram of sniffing before (blue) and after (red) odour infusion (mean ± SEM). (F) Correlation between LFP and sniff before (blue bar) and after (red bar) odour infusion (% of baseline values). *P < 0.05.

Septal sniff-modulated units express a higher degree of phase relation to the sniff cycle during higher sniff frequencies (see Figs 2B right and 3). Olfactory perception is intimately linked to the sniffing rate (Wesson *et al*., 2009) and one might hypothesise that the coupling between sniffing and spiking activity of MS/DB neurons is promoted by olfactory processing. We examined this hypothesis explicitly by infusing odorant through the nasal cannula and evaluating MS/DB neuronal firing. We compared spiking activity in the first 3 s after odour infusion to the preceding 3 s (Fig. 7A). Our choice of the post-infusion epoch duration was based on the finding that odour encoding of mitral cells in the OB occurs in dynamic timescales involving several sniffing cycles (Bathellier *et al*., 2008). The increase of sniffing frequency in the high theta range (9–12 Hz) for post-infusion epochs confirmed behaviourally that the odour was detected by the animals (Fig. 7E). To examine if the firing of fast-spiking neurons is linked to olfactory perception, we compared the polarity of all spikes in relation to the sniff phase before and after the odour infusion. Because different units show phase preference to different degrees (between 0 and 360°) we first normalised the polarity of all units. For this we aligned the peak firing frequency of each unit to 180° (see normalisation in Materials and methods). The averaged values of all normalised polarities prior to the odour infusion (Fig. 7B, blue trace) were compared with the averaged values of all normalised polarities after the odour infusion (Fig. 7B, red trace). The significant increase of phase polarity expressed by *post hoc* analysis of variance (*P* < 0.05; Newman–Keuls test) was confirmed with higher values of circular concentration coefficient *k* for post- (0.103 ± 0.011), compared with pre-infusion epochs (0.071 ± 0.008; *P* < 0.05, Watson–Williams test, Fig. 7B, inset). To determine the change of the fast-spiking patterns after odour infusion, we also compared the inter-spike interval with pre-infusion epochs. Increased numbers of inter-spike intervals were evident in the range 80–90 ms, of the high theta frequency of 10–12 Hz (Fig. 7D). These data show that the coupling of MS/DB neuronal activity to an animal∼s sniffing is augmented after olfactory stimulation.

Relevant olfactory input can reset the phase of limbic theta oscillation, and produce a short-lasting increase of coherence between theta rhythm and respiration. The septal region has the anatomical connectivity to integrate sniffing and theta because (i) it is connected with the pontine respiratory nuclei as well as with the olfactory cortex, and (ii) it innervates all limbic areas, and can induce simultaneous regulation of theta oscillations across multiple hippocampal and entorhinal areas. Thus, we aimed to determine if septal activity synchronises respiration and theta rhythm (although we are not aiming here to differentiate if odour-induced sniffing or fast sniffing frequency *per se* triggers this process). While the slow-spiking cells are constantly synchronised with the sniffing cycle, the fast-spiking and theta cells show temporal coupling. Such restricted coupling might be functionally linked to the coordination of two oscillators. Sniffing and limbic theta rhythm are oscillations with similar frequency in the range 5–12 Hz but with different peaks, suggesting that they are independent oscillators. The coupling between two independent oscillators could be coincidental or causal. To test the hypothesis that there is a causal relationship between theta and sniffing we examined if odour-potentiated phase-preference of fast-spiking parallels a change in the correlation between sniffing and hippocampal theta rhythm. We compared the changes in sniffing and LFP (band-pass filtered) for pre- and post-infusion epochs (Fig. 7C). Odour detection increased sniffing frequency (two-way anova, *F*_1,12_ = 9.12, *P* < 0.05, *n* = 8) in the range of 9–10 Hz for post-infusion epochs (Fig. 7E, Newman–Keuls test, *P* < 0.05). Importantly, the correlation between theta LFP and sniffing reached values of about 150%, compared with pre-infusion controls (*P* < 0.05, Mann*–*Whitney test, Fig. 7F).

### Hippocampal local field and single-unit activity relates to the sniff phase

**Fig. 8:**
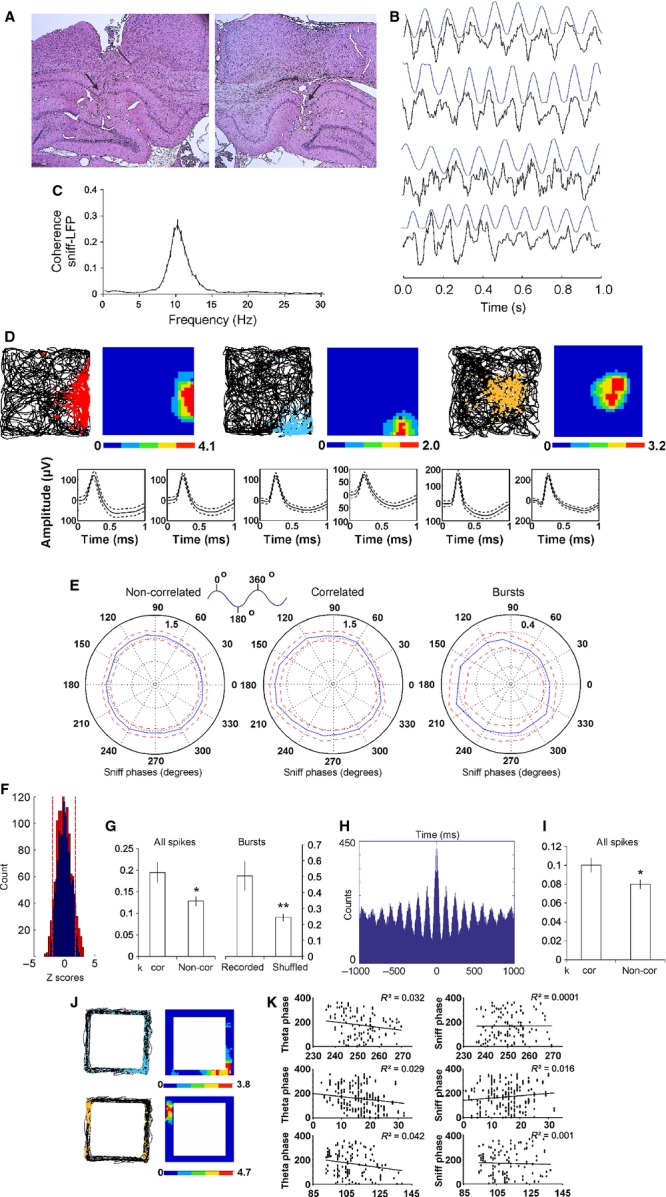
Hippocampal field and unit activity relates to sniff cycle. (A) Coronal histological sections from two rats where eight tetrodes were implanted in hippocampal CA1 region. The black arrow indicates the location of the tetrode tip. (B) Sample recordings of LFP and concurrent sniffing (blue) during epochs of high correlation. (C) Frequency histogram of the coherence between sniff and hippocampal LFP. (D) Sample place field maps of CA1 pyramidal neurons from three animals. Left map represents animal trajectory with spikes (red, blue, yellow), whereas on the right is positioned the corresponding firing rate map. Bottom: spike waveforms of the same place cells. (E) Average polar plots, relative to the sniff cycle for all CA1 place cells (mean ± SEM) during epochs with no sniff–LFP correlation (left) and during epochs with significant sniff–LFP correlation (middle). The polar plot on the right represents average burst polarity, relative to sniff cycle, for all CA1 place cells (mean ± SEM) for the entire recording sessions. (F) Distribution of Z-scores evaluates the significance of phase locking to the sniff cycle of hippocampal place cells for the correlated (red bars) and non-correlated group (blue bars). Significance probability associated with Z shows the relationship of the neuronal firing frequency across all phase bins (0–360°). Dashed red lines indicate the *P* = 0.05 significance threshold, and thus all values of Z to the right/left of these lines are significant at that confidence level. (G) Place cell circular statistics: left, average *k* values for correlated (cor) vs. non-correlated epochs (non-cor); right, average *k* values for recorded bursts vs. shuffled bursts. (H) Sample 1000-ms autocorrelogram of hippocampal theta-modulated interneuron. (I) Average *k* values for correlated (cor) vs. non-correlated epochs (non-cor) for interneurons. *P < 0.05. (J) Sample place field maps of CA1 pyramidal neurons from two animals after exploration of rectangular-shaped linear tracks. Left maps represents animal trajectory with spikes (blue, yellow), whereas on the right is the corresponding firing rate map. (K) Plot of theta phase (left) and sniff phase (right) vs. time for the overlaid spikes of runs in rectangular-shaped linear tracks for four representative place cells. The values of Pearson∼s correlation coefficient (*R*^2^) for each phase correlation are shown in the upper right corner of the plots.

The medial septum sends dense projections to the hippocampal formation and if hippocampal neurons are entrained by the MS/DB-generated theta rhythm, then correlations between theta and sniffing should reflect hippocampal place cell activity. To examine this proposal, we first compared the hippocampal LFP, recorded from the CA1 region of five rats (Fig. 8A), with sniff dynamics during pellet-chasing in an open square arena, to which the animals were habituated. Food-foraging behaviour evoked the frequent occurrence of epochs with a high correlation between the LFP and sniffing (Fig. 8B) with a peak of coherence in the theta range, with maximum at 10 Hz (0.26 ± 0.02, Fig. 8C). We next examined if the spiking of hipppocampal neurons reflects the coupling of the LFP to sniffing during epochs of significant theta–sniff correlation.

We compared the phase relation of 52 place cells and 38 interneurons to the sniff-cycle between epochs with significant (*P* < 0.05) and epochs with non-significant (*P* > 0.05) theta–sniff correlation (see Materials and methods). The significant and non-significant correlation epochs were equally distributed in the maze without relation to an animal∼s trajectory. The significant correlation epochs were predominantly expressed during locomotion of 10–20 cm/s, which was the main running speed of the animals the during pellet chasing task. The identification of place cells was based on their spike shape, firing frequency and spatial properties (Fig. 8D). Because of their low firing frequency, the recording of one unit provides an insufficient number of spikes that can be compared with circular statistics to the shuffled data (Fig. S4). To increase the sampling number, we averaged the sniff polarity for all place cells for non-significant vs. significant correlation epochs. The non-correlation group showed no preference of the place cell spiking across the sniff cycle (Fig. 8E, left). The values of circular coefficient *k* for the non-correlation group did not show statistical significance compared with the *k* values of the shuffled place cells’ spikes (*P* = 0.28, Watson–Williams test). However, the correlation group revealed a preference of spiking for the range 180–240° (Fig. 8E, middle) and this observation was confirmed by a significantly higher circular coefficient *k* compared with non-correlation group (*P* < 0.05, Watson–Williams test, Fig. 8G, left). Importantly, Z-scores comparison of the place cell firing frequency for correlation (Fig. 8F, red bars) vs. non-correlation group (Fig. 8F, blue bars) showed a greater number of Z-scores above the significance threshold (marked with red dashed lines) for the sniff–theta correlation group. As the highest degree of sniff–theta coherence occurs at 10 Hz (Fig. 8C), and at this frequency place cells spike predominantly in bursts (complex-spike units entrained by theta), our next intention was to evaluate the sniff polarity for the bursts from all recorded epochs. The recorded bursts also showed a preference for sniff phase in the range 180–240° (Fig. 8E, right), with *k* significantly higher than the shuffled bursts (*P* < 0.01, Watson–Williams test, Fig. 8G, right). Therefore, place activity is tuned to the trough of the sniff cycle and this might be mediated through short-lasting synchronisation of local theta oscillators to the respiratory rate. Similarly, the sniff-phase polarity of theta-modulated hippocampal interneurons (Fig. 8H) was dependent on the degree of the sniff–LFP correlation. The significantly higher value of the circular coefficient *k* compared with the non-correlated epochs (*P* < 0.05, Watson–Williams test, Fig. 8I) suggests that rhythmic activity of these neurons is temporally reset by incoming inputs. Phase precession is an intrinsic feature of hippocampal neurons (O∼Keefe & Recce, 1993) and, despite the transient coupling between sniff and theta, the phase precession from place field recordings in linear tracks (Fig. 8J) correlated with theta (Fig. 8K, left, Pearson∼s *r* = −0.171, *P* < 0.01; *n* = 42) but not sniff oscillation (Fig. 8K, right; Pearson∼s *r* = 0.012, *P* = 0.16; *n* = 38). This result shows that the sniffing rate is synchronised for brief episodes with the hippocampal LFP, which are insufficient to form persistent coupling between sniff phase and place cell spiking.

### Muscimol abolishes in parallel fast sniffing and high theta but preserves their relationship

**Fig. 9:**
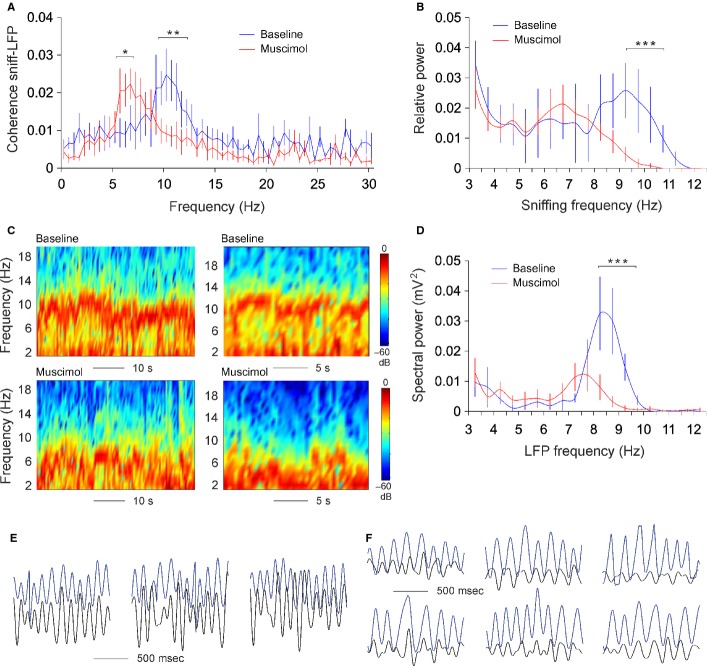
Muscimol abolishes fast sniffing and high theta but preserves their relation. *P < 0.05; **P < 0.01. (A) Frequency histogram of the coherence between sniff and hippocampal LFP before (blue) and after (red) muscimol injection. (B) Frequency histogram of sniffing frequency before (blue) and after (red) muscimol injection. ***P < 0.001. (C) Colour-coded power spectrograms of hippocampal oscillation before (upper panels) and after (lower panels) muscimol injection. (D) Frequency histogram of local field potential before (blue) and after (red) muscimol injection. (E) Sample recordings of band-pass filtered local field potential (black) and concurrent sniffing (blue) during epochs of baseline high correlation. (F) Sample recordings of local field potential (black) and concurrent sniffing (blue) during epochs of post-injection high correlation.

Odour-induced sniffing promotes the correlation between sniffing and hippocampal LFP (Fig. 7), but the evidence showing the role of MS/DB in both sniffing rate and theta rhythm is insufficient. The coherence between sniffing and theta is shown to follow a linear pattern in relation to frequency, where the sniffs are timed to maintain a preferred latency relationship with theta rhythm during different theta–sniff frequencies (Macrides *et al*., 1982). To demonstrate the functional role of MS/DB in the frequency control of the sniffing–theta coherence, we performed unilateral muscimol microinfusions to attenuate septal activity pharmacologically (Shirvalkar *et al*., 2010; Brandon *et al*., 2011). The inactivation of medial septum in rats causes disruption of theta oscillations in the hippocampus (Rawlins *et al*., 1979; Mizumori *et al*., 1990) and impairs hippocampal-dependent memory (Winson, 1978; Givens & Olton, 1994). Fifteen minutes after muscimol injection, we monitored sniffing and concurrent local field activity in CA1 stratum radiatum from dorsal hippocampus. Interestingly, the coherence between sniffing and LFP was preserved, although it was shifted to a lower frequency range with peak of 6 Hz, compared with a pre-infusion peak of 10 Hz (Fig. 9A). After injection of muscimol into the MS/DB, the fast component of the sniffing frequency (9–12 Hz) was strongly attenuated, compared with baseline controls (Fig. 9B, anova, *F*_1,8_ = 13.75, *P* < 0.01, *n* = 4). In parallel, the hippocampal recordings showed a clear decrease of the local spectral power amplitude in the range of theta frequency (Fig. 9C and D). The peak of post-injection theta frequency was 7.5 Hz, compared with 8.5 Hz of pre-injection baseline (Fig. 9D, anova, *F*_1,8_ = 16.14, *P* < 0.01, *n* = 4). The coherent episodes between sniffing and theta from baseline recordings (Fig. 9E) were preserved after muscimol injection, although the amplitude and frequency of theta as well as the sniffing frequency were reduced (Fig. 9F). Muscimol injection also abolished the odour-triggered augmentation of the correlation between sniffing and LFP with no statistical difference between the pre- and post-infusion epochs correlation (*P* = 0.37, *t*-test, *n* = 4). These data show that muscimol-induced inhibition of MS/DB activity triggers parallel shifts of sniffing rate and hippocampal theta.

## Discussion

We provide novel evidence here that a substantial population of neurons in the MS/DB discharge in synchrony with an animal∼s sniffing. Single-unit recordings in chronically implanted rats revealed that slow-spiking units are phase-locked to the sniffing cycle, while the spike trains and bursts of fast spiking and theta units expressed a preference to the sniff phase. Correlation analyses of septal inter-spike intervals and sniffing oscillations showed significant peaks of correlation for all cell types. We also demonstrate that inhibition of septal theta neurons reduces the frequency of sniffing-theta coherence, while odour infusion augments the correlation between sniffing and hippocampal theta rhythm.

Synchronous oscillatory activity functionally links remote neuronal populations or brain areas, providing a temporal window for transient communication (Fries, 2005). Olfactory neuronal and local field activity is locked to respiration (Cang & Isaacson, 2003; Fantana *et al*., 2008), and respiration can synchronise with hippocampal theta rhythm (Macrides *et al*., 1982). As a result, hippocampal theta and OB oscillations are correlated during performance of olfactory discrimination tasks (Kay, 2005; Martin *et al*., 2007). The activity in limbic structures has been linked to respiration (Harper *et al*., 1998), suggesting that phase synchronisation between respiration and limbic rhythms may have a crucial role in the formation of hippocampal-dependent memory for odours. Thus, the sniff cycle may act as a coherent ‘timing unit’ for olfactory and hippocampal systems (Buonviso *et al*., 2006).

### Functional relation of MS/DB to respiratory circuitry and olfactory processing

The respiratory rhythm is generated within the ventrolateral side of the brainstem and is distributed between the pontine, ventral and dorsal respiratory groups (Onimaru & Homma, 2003; Smith *et al*., 2007). The pontine respiratory group (including the pedunculopontine tegmental and laterodorsal tegmental nuclei) sends ascending afferents to MS/DB complex (Cornwall *et al*., 1990; Woolf *et al*., 1990), the region of our interest. Concurrently, MS/DB receives indirect olfactory inputs via the piriform cortex and hypothalamus (Price & Powell, 1970; Carnes *et al*., 1990), allowing the septal region to integrate the motor and the sensory components of olfactory perception. Importantly, neuronal activity in anterior piriform cortex is phase-locked to the sniff cycle (Rennaker *et al*., 2007). Here, we show that 52.4% of recorded units in MS/DB (175 of 334) relate their spiking to respiration. Slow-spiking units demonstrate a strict phase-locking to the sniff cycle, whereas the fast-spiking and theta units entrain their burst with respect to sniff phase. The spiking of all three groups is correlated with the sniffing rate. The peaks of the correlation are either positive or negative, revealing that each neuron shows an individual preference for the sniff phase. Interestingly, slow-spiking units are characterised by two peaks of correlation where the second peak is about −530 ms from time 0 (Fig. 6C). Thus, sniff and theta couple their activity in windows of four sniff cycles on average. Over a similar time frame (−550 ms from time 0), the negative–positive correlation peak sequences of theta units are distributed (Fig. 6E), representing the frequency of theta-burst and subsequent inter-burst pauses. These data show that slow-spiking and theta units are functionally related. Both groups fire regularly (in spikes or bursts, respectively) with correlation peaks of < −30 ms to time 0. Both neuronal types fire with a higher correlation during fast sniffing (9–12 Hz), and this frequency precisely defines the range of coherence between the hippocampal LFP and sniffing (Fig. 8C). Fast-spiking units fire with irregular bursts and show a high degree of correlation to the sniff rate with a temporal delay of about −100 ms. This timing is sufficient for polysynaptic signal processing, following the anatomical and physiological connectivity between olfactory structures and MS/DB (Barkai & Hasselmo, 1997; Linster & Hasselmo, 2000). The correlations between septal inter-spike intervals and sniffing revealed significant but relatively low values of about ± 0.03. The weak correlation shows that MS/DB neuronal activity is not solely involved in olfactory processing but rather integrates processed respiratory signals. Additionally, the timing of neuronal activity in the OB relative to the onsets of airflow into the nasal cavity can vary by hundreds of milliseconds depending on the odorants in the inhaled air (Macrides & Chorover, 1972; Cury & Uchida, 2010; Carey & Wachowiak, 2011), which can also lead to noise in the sniff-spiking correlations. Early work has proposed the influence of respiration on theta frequency oscillations in the OB and piriform cortex (Eeckman & Freeman, 1990; Barrie *et al*., 1996). The olfactory processing involves sensory (olfactory) and motor (respiratory) signal integrity, and thus MS/DB might mediate processed olfactory sensorimotor inputs. Our data support this hypothesis, demonstrating that olfactory perception induced by odorant stimulation augments the degree of neuronal polarity to the sniff phase (Fig. 7B). We also aimed to find if the olfactory-induced sniff phase preference of septal neurons reflects the functional activity of MS/DB output regions.

### Integration of sniff rate and theta frequency by septo-hippocampal circuitry

A major MS/DB output targets the hippocampal system (Amaral & Kurz, 1985; Wainer *et al*., 1985). Lesions of the septal inputs impair hippocampal theta rhythm by reducing its rate and decreasing its power (Rawlins *et al*., 1979; Buzsáki *et al*., 1983; Lee *et al*., 1994; Bassant *et al*., 1995). Septal inactivation decreases the firing rate of hippocampal neurons and reduces their ability to spike in trains (Leutgeb & Mizumori, 1999; Koenig *et al*., 2011). Current theories on the generation of theta oscillations in the hippocampal formation attribute a central role to interconnected inhibitory GABA neurons, either as oscillators or as resonators to theta activity (Buzsáki, 2002). In agreement with earlier studies (Macrides *et al*., 1982; Kay, 2005) we show here that hippocampal theta rhythm increases its correlation with respiratory oscillations during odour-induced epochs of faster sniffing in the range 9–12 Hz (Fig. 7C). The spiking of place cells depends on a number of variables, including the position of the animal, and the theta phase (O∼Keefe & Recce, 1993; Harris *et al*., 2002; Mehta *et al*., 2002). Hippocampal theta amplitude and frequency depend on locomotor behaviour (Vanderwolf, 1969; Geisler *et al*., 2007), which entrains the firing rate of hippocampal place cells as a function of an animal∼s speed (McNaughton *et al*., 1983; Wiener *et al*., 1989; Czurkó *et al*., 1999). Rodent locomotion strongly interferes with sniffing behaviour and anatomically this is related to the activity of pontine cholinergic structures (the pedunculopontine tegmental and laterodorsal tegmental nuclei), which converge inputs from basal ganglia and respiratory circuitry (Kubin & Fenik, 2004; Mena-Segovia *et al*., 2004). Thus, we explored if hippocampal neurons could reflect the sniffing phase during an animal∼s locomotion in a pallet-chasing task. Indeed, we found that place cells burst with a preference for the sniff phase in the range 180–240° (Fig. 8E). This phase-preference precisely matches the ability of CA1 place cells to fire with highest frequency on the trough of theta in the range 180–270° (O∼Keefe & Recce, 1993; Mehta *et al*., 2002). The coupling of place cell spiking to the sniff phase in our experiments was expressed during epochs of significant sniff–LFP correlations. Such a relationship has been proposed by observations of hippocampal neurons, recorded from partial complex seizure patients, which showed an overall rate correlation with the respiratory cycle, with cells discharging on a breath-by-breath relationship (Frysinger & Harper, 1989).

The phase link between sniffing and hippocampal theta rhythm has been shown to vary linearly with frequency (Macrides *et al*., 1982). If both oscillations are functionally related to MS/DB, then inactivation of the septal region should evoke concurrent changes in both frequencies. To test if MS/DB reflects the frequency of both respiratory and hippocampal local field oscillations, we inhibited septal activity by injection of a GABAergic agonist (muscimol). We found that both sniffing and theta rhythm underwent a low-frequency shift: both oscillators lost their upper theta range (9–12 Hz). Importantly, the coherence between them was preserved in the range of 6 Hz (Fig. 9). The preserved information processing between both oscillators is supported by the finding that inhalation-coupled transient activity in OB is invariant to respiration frequency (Cury & Uchida, 2010). The muscimol experiment demonstrated that MS/DB plays a crucial role in resetting both theta and respiration oscillations and particularly their fast frequency (9–12 Hz) bands, which are characteristic features of the odour-induced sniff–theta correlation. The fact that the sniffing frequency was concurrently decreased proposes bidirectional connectivity between septal and ponto-medullar neuronal groups (both of which are cholinergic). Respiration drives directly or indirectly (through OB and piriform corthex) septal neurons, while medial septum regulates the frequency of sniffing. The partial inhibition of MS/DB led to a synchronised decrease of theta and sniffing frequencies. There is also the possibility that MS/DB is just one of the structures that mediate the sniff–theta coupling, particularly in the slow frequencies. Other candidates involved in this network might be nucleus basalis magnocellularis (also a cholinergic structure) as well as the hippocampal formation itself. Nucleus basalis of the basal forebrain forms extensive cholinergic projections to the cortex (Woolf *et al*., 1983; Casamenti *et al*., 1986) and it plays a role in arousal, sensorimotor integration and locomotion (Wenk, 1997; Wrenn & Wiley, 1998). Inactivation of nucleus basalis affects the cortical oscillatory synchrony (Buzsáki *et al*., 1988; Detari *et al*., 1999; Sarter & Bruno, 2000). Nucleus basalis also receives inputs from pediculopontine tegmental nucleus (Semba *et al*., 1988), which could be a potential bypass for sniff–theta coupling, for the low (0–5 Hz) frequencies. Overall, the cholinergic circuitry of ponto-medullar nuclei and basal forebrain controls both limbic oscillations and exploratory behaviour, thus enabling a functional synchronisation of different brain oscillators.

In summary, our data suggest that the septo-hippocampal axis integrates respiratory rate and limbic theta rhythm. This temporal coupling incorporates the integration of intrinsic theta and extrinsic sensorimotor signals on each theta cycle. The functional significance of our data addresses the assembly organisation of hippocampal neurons, which is crucially dependent on the relationship between global theta frequency, the oscillation frequency of single neurons and the sensorimotor theta timescale (Buzsáki, 2010).

## Abbreviations

LFP: local field potential
MS/DB: medial septum/diagonal band of Broca
OB: olfactory bulb

## Supporting Information

Fig. S1. Slow-spiking activity for the main sniffing frequencies. Fig. S2. Fast-spiking activity for the main sniffing frequencies. Fig. S3. Theta activity for the main sniffing frequencies. Fig. S4. Place cells∼ relation to sniffing parameters.
