# Supplementary material for: Respiratory cycle entrainment of septal neurons mediates the fast coupling of sniffing rate and hippocampal theta rhythm
Source: Eur J Neurosci. 2014 Aug 20;39(6):957–74. doi: 10.1111/ejn.12449 (PMC4165309; doi:10.1111/ejn.12449)
Supplement: Supplementary file 1 [file ejn0039-0957-SD1.pdf]

SUPPORTING INFORMATION - SUPPLEMENTARY FIGURES – Tsanov et al., 2013

Figure S1. Slow-spiking activity for the main sniffing frequencies. (A) Sample polar plots, showing firing rate in Hz (dotted circles), relative to the sniff cycle for eight slow-spiking units. The peak-to-peak distance of one sniff cycle is considered as 0-360° where the trough is 180°. Each panel shows the phase parameters for recorded spikes (red, left) and shuffled (blue, right) data. (B) Sample polar plots, relative to the sniff cycle for eight slow-spiking units in relation to the sniffing frequency: left panels – 0-5Hz, middle panels – 5-9Hz, right panels – 9-12Hz. Red traces represent recorded, while blue traces represent shuffled spikes.

Figure S2. Fast-spiking activity for the main sniffing frequencies. (A) Sample polar plots, showing firing rate in Hz, relative to the sniff cycle for four fast-spiking units for recorded spikes (red, left) and shuffled (blue, right) data. (B) Sample polar plots, relative to the sniff cycle for the same units for spike trains. (C) Sample polar plots, relative to the sniff cycle for same units for bursts. Red traces represent recorded, while blue traces represent shuffled spikes. (D) Sample polar plots, relative to the sniff cycle for the same fast-spiking units in relation to the sniffing frequency: left panels – 0-5Hz, middle panels – 5-9Hz, right panels – 9-12Hz. Red traces represent recorded, while blue traces represent shuffled spikes.

Figure S3. Theta activity for the main sniffing frequencies. (A) Sample polar plots, showing firing rate in Hz, relative to the sniff cycle for four theta units for recordes spikes (red, left) and shuffled (blue, right) data. (B) Sample polar plots, relative to the sniff cycle for the same units for spike trains. (C) Sample polar plots, relative to the sniff cycle for same units for bursts. Red traces represent recorded, while blue traces represent shuffled spikes. (D) Sample polar plots, relative to the sniff cycle for the same theta units in relation to the sniffing frequency: left panels – 0-5Hz, middle panels – 5-9Hz, right panels – 9-12Hz.

Figure S4. Place cells' relation to sniffing parameters. (A) Polar plots, relative to the sniff cycle for recorded spikes of six CA1 place cells during epochs with significant sniff-LFP correlation (left) and during epochs with no sniff-LFP correlation (right). (B) Polar plots, relative to the sniff cycle for the same place cells with shuffled spikes during epochs with significant sniff-LFP correlation (left) and during epochs with no sniff-LFP correlation (right).

Figure S1:

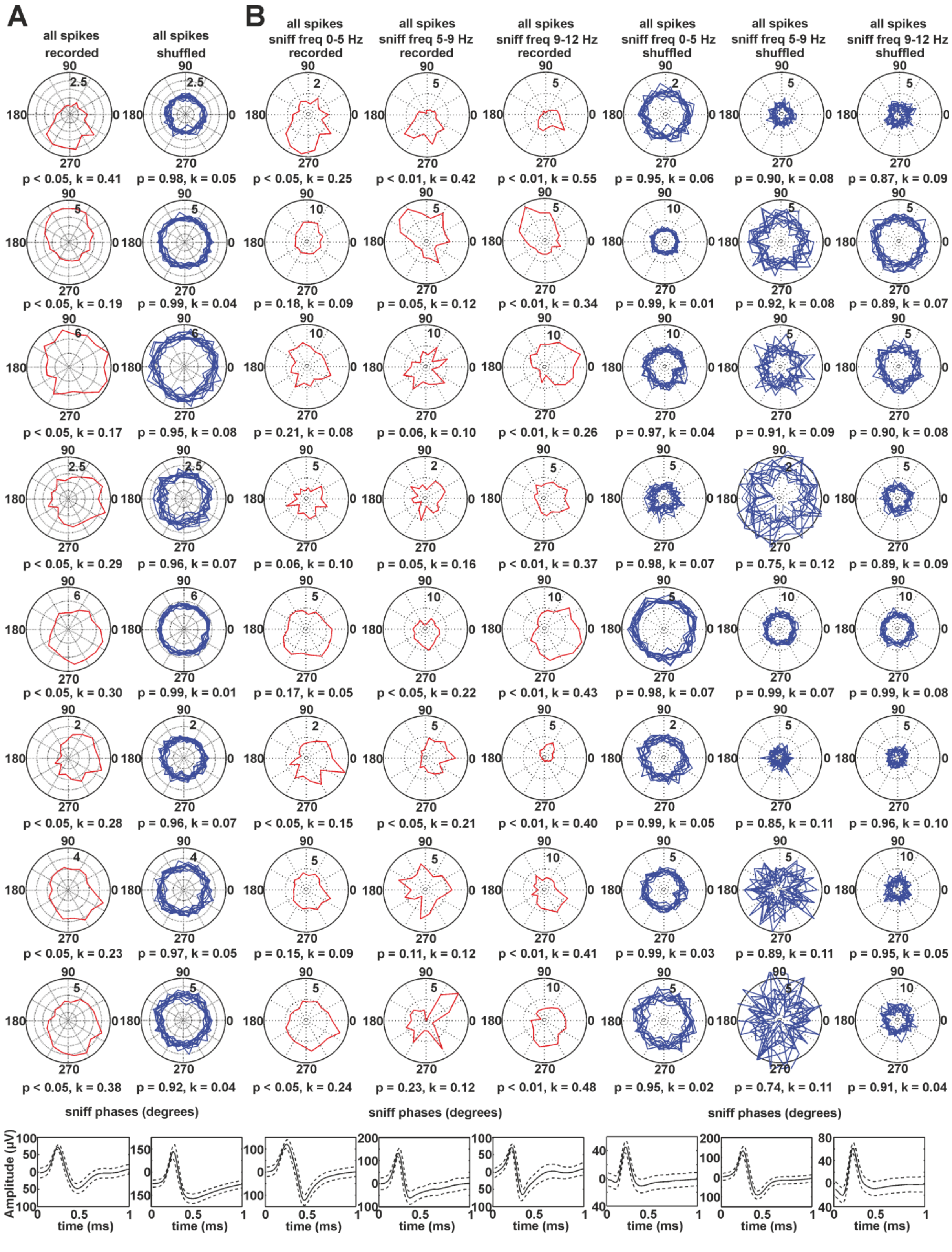
A
B
all spikes recorded
all spikes shuffled
all spikes sniff freq 0-5 Hz recorded
all spikes sniff freq 5-9 Hz recorded
all spikes sniff freq 9-12 Hz recorded
all spikes sniff freq 0-5 Hz shuffled
all spikes sniff freq 5-9 Hz shuffled
all spikes sniff freq 9-12 Hz shuffled
p < 0.05, k = 0.41
p = 0.98, k = 0.05
p < 0.05, k = 0.25
p < 0.01, k = 0.42
p < 0.01, k = 0.55
p = 0.95, k = 0.06
p = 0.90, k = 0.08
p = 0.87, k = 0.09
p < 0.05, k = 0.19
p = 0.99, k = 0.04
p = 0.18, k = 0.09
p = 0.05, k = 0.12
p < 0.01, k = 0.34
p = 0.99, k = 0.01
p = 0.92, k = 0.08
p = 0.89, k = 0.07
p < 0.05, k = 0.17
p = 0.95, k = 0.08
p = 0.21, k = 0.08
p = 0.06, k = 0.10
p < 0.01, k = 0.26
p = 0.97, k = 0.04
p = 0.91, k = 0.09
p = 0.90, k = 0.08
p < 0.05, k = 0.29
p = 0.96, k = 0.07
p = 0.06, k = 0.10
p = 0.05, k = 0.16
p < 0.01, k = 0.37
p = 0.98, k = 0.07
p = 0.75, k = 0.12
p = 0.89, k = 0.09
p < 0.05, k = 0.30
p = 0.99, k = 0.01
p = 0.17, k = 0.05
p < 0.05, k = 0.22
p < 0.01, k = 0.43
p = 0.98, k = 0.07
p = 0.99, k = 0.07
p = 0.99, k = 0.08
p < 0.05, k = 0.28
p = 0.96, k = 0.07
p < 0.05, k = 0.15
p < 0.05, k = 0.21
p < 0.01, k = 0.40
p = 0.99, k = 0.05
p = 0.85, k = 0.11
p = 0.96, k = 0.10
p < 0.05, k = 0.23
p = 0.97, k = 0.05
p = 0.15, k = 0.09
p = 0.11, k = 0.12
p < 0.01, k = 0.41
p = 0.99, k = 0.03
p = 0.89, k = 0.11
p = 0.95, k = 0.05
p < 0.05, k = 0.38
p = 0.92, k = 0.04
p < 0.05, k = 0.24
p = 0.23, k = 0.12
p < 0.01, k = 0.48
p = 0.95, k = 0.02
p = 0.74, k = 0.11
p = 0.91, k = 0.04
sniff phases (degrees)
sniff phases (degrees)
sniff phases (degrees)
Amplitude (µV)
time (ms)

Figure S2:

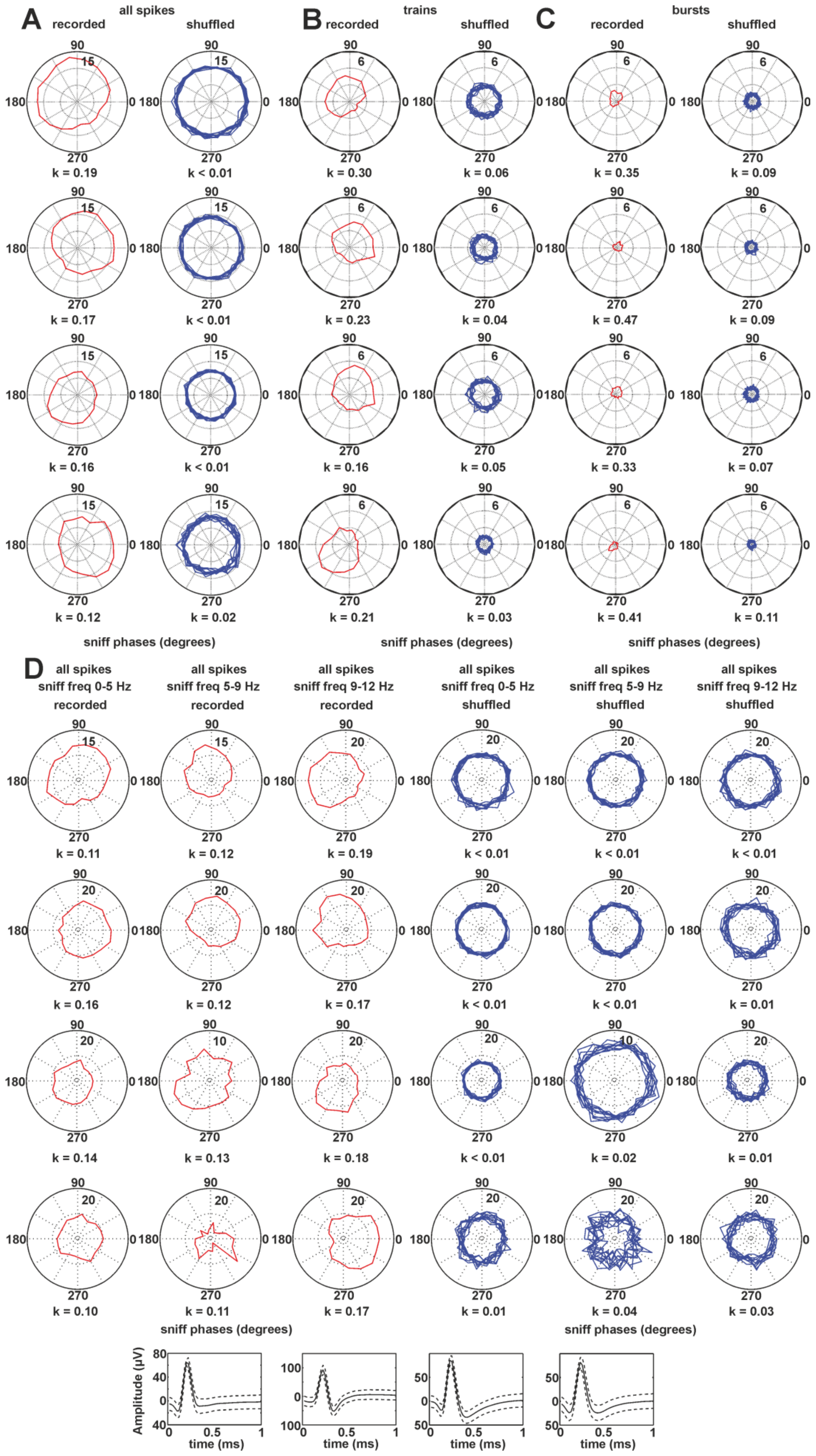
A
all spikes
recorded
shuffled
B
trains
recorded
shuffled
C
bursts
recorded
shuffled
k = 0.19
k < 0.01
k = 0.30
k = 0.06
k = 0.35
k = 0.09
k = 0.17
k < 0.01
k = 0.23
k = 0.04
k = 0.47
k = 0.09
k = 0.16
k < 0.01
k = 0.16
k = 0.05
k = 0.33
k = 0.07
k = 0.12
k = 0.02
k = 0.21
k = 0.03
k = 0.41
k = 0.11
sniff phases (degrees)
sniff phases (degrees)
sniff phases (degrees)
D
all spikes sniff freq 0-5 Hz recorded
all spikes sniff freq 5-9 Hz recorded
all spikes sniff freq 9-12 Hz recorded
all spikes sniff freq 0-5 Hz shuffled
all spikes sniff freq 5-9 Hz shuffled
all spikes sniff freq 9-12 Hz shuffled
k = 0.11
k = 0.12
k = 0.19
k < 0.01
k < 0.01
k < 0.01
k = 0.16
k = 0.12
k = 0.17
k < 0.01
k < 0.01
k = 0.01
k = 0.14
k = 0.13
k = 0.18
k < 0.01
k = 0.02
k = 0.01
k = 0.10
k = 0.11
k = 0.17
k = 0.01
k = 0.04
k = 0.03
sniff phases (degrees)
sniff phases (degrees)
Amplitude (µV)
time (ms)
time (ms)
time (ms)
time (ms)

Figure S3:

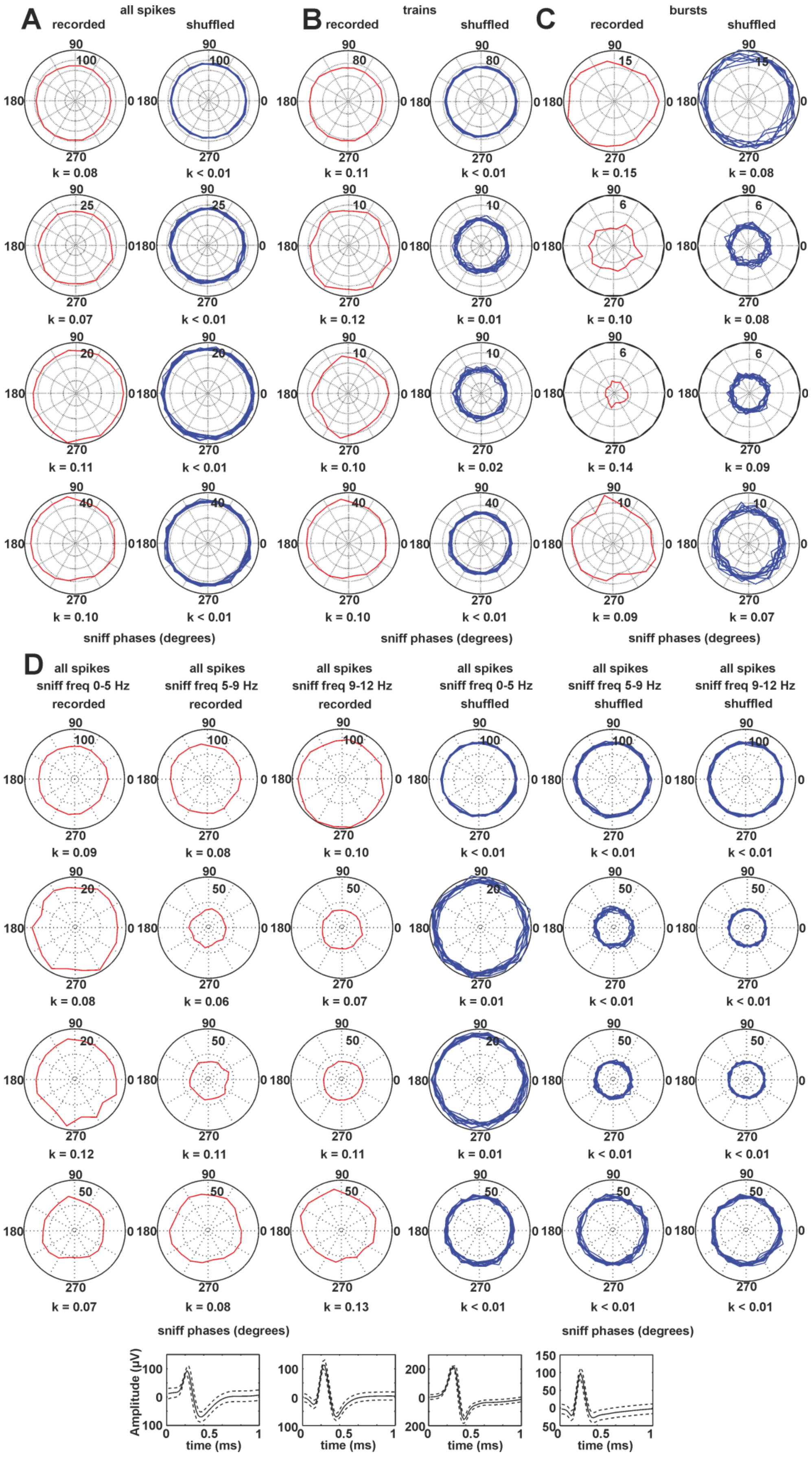
A
all spikes
recorded
shuffled
B
trains
recorded
shuffled
C
bursts
recorded
shuffled
k = 0.08
k < 0.01
k = 0.11
k < 0.01
k = 0.15
k = 0.08
k = 0.07
k < 0.01
k = 0.12
k = 0.01
k = 0.10
k = 0.08
k = 0.11
k < 0.01
k = 0.10
k = 0.02
k = 0.14
k = 0.09
k = 0.10
k < 0.01
k = 0.10
k < 0.01
k = 0.09
k = 0.07
sniff phases (degrees)
D
all spikes
sniff freq 0-5 Hz
recorded
all spikes
sniff freq 5-9 Hz
recorded
all spikes
sniff freq 9-12 Hz
recorded
all spikes
sniff freq 0-5 Hz
shuffled
all spikes
sniff freq 5-9 Hz
shuffled
all spikes
sniff freq 9-12 Hz
shuffled
k = 0.09
k = 0.08
k = 0.10
k < 0.01
k < 0.01
k < 0.01
k = 0.08
k = 0.06
k = 0.07
k = 0.01
k < 0.01
k < 0.01
k = 0.12
k = 0.11
k = 0.11
k = 0.01
k < 0.01
k < 0.01
k = 0.07
k = 0.08
k = 0.13
k < 0.01
k < 0.01
k < 0.01
sniff phases (degrees)
Amplitude (μV)
time (ms)

Fig S4:

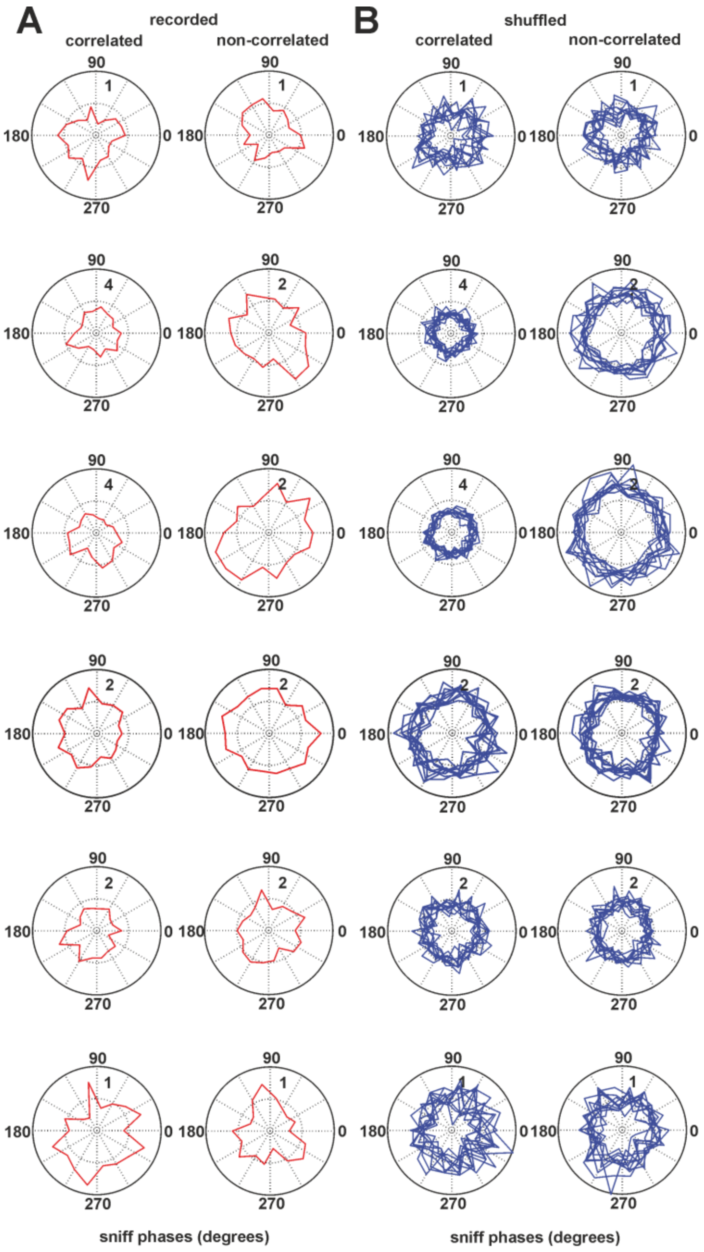

A
recorded
correlated
non-correlated
B
shuffled
correlated
non-correlated
90
180
0
270
sniff phases (degrees)
sniff phases (degrees)
